# Reductions in tyrosine levels are associated with thyroid hormone and catecholamine disturbances in sepsis

**DOI:** 10.1186/2197-425X-3-S1-A686

**Published:** 2015-10-01

**Authors:** W Khaliq, DT Andreis, A Kleyman, M Gräler, M Singer

**Affiliations:** Bloomsbury Institute of Intensive Care Medicine, University College London, London, United Kingdom; Dipartimento di Fisiopatologia Medico-Chirurgica e dei Trapianti, Università degli Studi di Milano, Milan, Italy; Department of Anesthesiology and Intensive Care Medicine, University Hospital Jena, SG Sepsis Research, Jena, Germany

## Introduction

The magnitude of fall in thyroid hormone and rise in catecholamine levels are prognostic in critical illness (1, 2). These may be linked, as the amino acid tyrosine is converted by tyrosine hydroxylase to L-DOPA, and thence to dopamine, norepinephrine and epinephrine, whereas thyroxine (T4) and tri-iodothyronine (T3) are formed by the iodination of tyrosine residues in thyroglobulin. We used our well-characterized 72 h fluid-resuscitated rat model of faecal peritonitis, where accurate prognostication can be made as early as 6 h (3), to explore any relationship.

## Objectives

To determine if any association exists between early changes in circulating tyrosine, thyroid hormone and catecholamine levels, and eventual outcome in this rat peritonitis model.

## Methods

Awake, instrumented yet fully mobile male Wistar rats (325 ± 15 g) received an i.p. injection of 4µl/g faecal slurry. Fluid resuscitation (50:50 mix of 5% glucose/Hartmann's; 10 ml/kg/h) was commenced at 2 h. At 6 h, an echo-measured heart rate cut-off of 460 bpm was used to classify animals into predicted survivors or non-survivors. Blood samples were also taken for measurement of plasma L-tyrosine (mass spectrometry), thyroid hormone and catecholamine levels (ELISA). Control animals were treated identically except for slurry injection. Results were analysed using two-way ANOVA and post-hoc testing and considered statistically significant when p < 0.05.

## Results

Predicted survivor and non-survivor septic animals were clinically indistinguishable at 6 h. Tyrosine levels were similarly low and catecholamine levels similarly elevated in both septic subgroups (p < 0.05), whereas non-survivors had significantly lower levels of T3 and T4 (p < 0.05).

## Conclusions

Sepsis resulted in significant early reductions in circulating tyrosine levels. The elevation of plasma catecholamines suggests increased hydroxylation of tyrosine, while reductions in thyroid hormone levels may be due to reduced tyrosine iodination. This relationship has not, to our knowledge, been previously described in sepsis and warrants further investigation.

## Grant Acknowledgment

ESICM Basic Science Award, Intensive Care Foundation (UK), NIHR.

**Table 1:** Table 1

	Control (n=6)	Predicted survival (n=6)	Predicted non-survival (n=6)
L-Tyrosine (µmol/L)	90.2 ± 3.3	58.8 ± 3.3 ^a^	62.7 ± 1.3 ^a^
T3 (pg/mL)	3.20 ± 0.41	2.77 ± 0.44	1.27 ± 0.25 ^a, b^
T4 (ng/mL)	9.20 ± 0.75	7.19 ± 0.50 ^a^	5.03 ± 1.02 ^a, b^
TSH (ng/mL)	1.25 ± 0.30	1.55 ± 0.21	1.64 ± 0.15
Adrenaline (ng/mL)	8.56 ± 0.42	9.44 ± 0.23	10.3 ± 0.18
Noradrenaline (ng/mL)	1.60 ± 0.25	3.21 ± 0.23 ^a^	2.98 ± 0.27 ^a^
***Data shown as median ± SE;*** ^***a***^ ***p < 0.05 versus control,*** ^***b***^ ***p < 0.05 versus survivors***			

## References

[1] Bacci V (1982). J Clin Endocrinol Metab.

[2] Hahn P (1995). Shock.

[3] Rudiger A (2013). Clin Sci.

